# Hypoxia, Epithelial-Mesenchymal Transition, and TET-Mediated Epigenetic Changes

**DOI:** 10.3390/jcm5020024

**Published:** 2016-02-04

**Authors:** Shih-Han Kao, Kou-Juey Wu, Wen-Hwa Lee

**Affiliations:** 1 Research Center for Tumor Medical Science and Graduate Institute of Cancer Biology, China Medical University, Taichung 40402, Taiwan; shkao@mail.cmu.edu.tw; 2 Institute of Clinical Medicine, China Medical University, Taichung 40402, Taiwan

**Keywords:** TET, 5hmC, DNA demethylation, hypoxia, HAP

## Abstract

Tumor hypoxia is a pathophysiologic outcome of disrupted microcirculation with inadequate supply of oxygen, leading to enhanced proliferation, epithelial-mesenchymal transition (EMT), metastasis, and chemo-resistance. Epigenetic changes induced by hypoxia are well documented, and they lead to tumor progression. Recent advances show that DNA demethylation mediated by the Ten-eleven translocation (TET) proteins induces major epigenetic changes and controls key steps of cancer development. TET enzymes serve as 5mC (5-methylcytosine)-specific dioxygenases and cause DNA demethylation. Hypoxia activates the expression of TET1, which also serves as a co-activator of HIF-1α transcriptional regulation to modulate HIF-1α downstream target genes and promote epithelial-mesenchymal transition. As HIF is a negative prognostic factor for tumor progression, hypoxia-activated prodrugs (HAPs) may provide a favorable therapeutic approach to lessen hypoxia-induced malignancy.

## 1. Introduction

**Figure 1:**
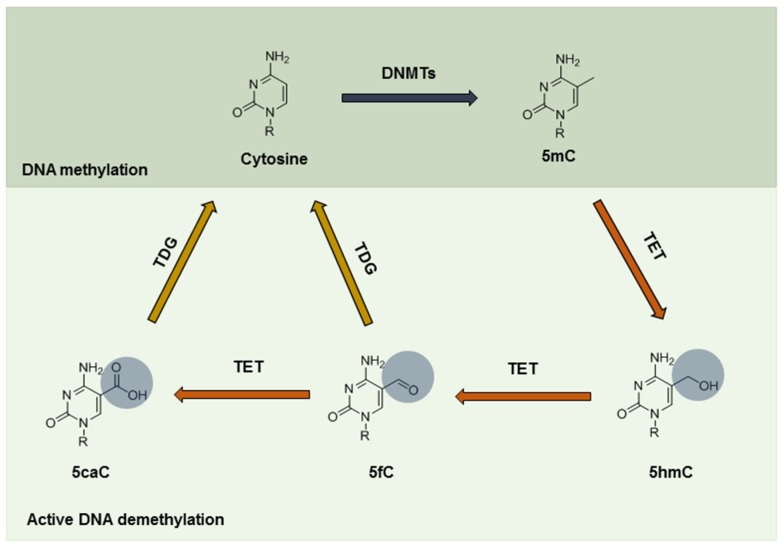
Chemical structures of TET-oxidized products. The DNA base cytosine can be methylated as epigenetic regulation at the DNA level by DNMTs. DNA methylation affects transcription and governs genomic stability during development as well as cancer progression. The methylated product (5-methylcytosine, 5mC) can be reversed actively by a set of enzymes, TET proteins and TDG back to cytosine. The intermediate products include: 5-hydroxymethylcytosine (5hmC), 5-formylcytosine (5fC), and 5-carboxylcytosine (5caC). The amount of 5hmC serves as a marker to indicate the status of DNA demethylation. Loss of 5hmC is often seen in cancers, particularly in myeloid and leukemia. TET: the Ten-eleven translocation family protein; DNMT: DNA methyltransferase; TDG: thymine-DNA glycosylase.

Low oxygen, or hypoxia, is a hallmark of tumor mass formation. Studies have indicated that hypoxia heralds the epithelial-mesenchymal transition (EMT), metastasis, angiogenesis, and negative clinical outcome [1,2]. Tumor cells exposed to hypoxia may experience a profiling change at the epigenetic level, leading to overall aggressiveness. DNA methylation/demethylation epigenetically governs transcription, genome stability, and development by associating with DNA imprinting, X-chromosome inactivation, suppression of repetitive elements, and carcinogenesis. Aberrant methylation of promoter regions, for example, leads to gene silencing, particularly at the promoter of tumor suppressors [3]. A dysregulated mechanism in cancer also involves the process of methyl group removal, which can be a passive or active process. While passive DNA demethylation results from inhibition of methyltransferases during cell division and replication by replacement with unmethylated cytosine, active demethylation needs direct enzymatic removal of the methyl group [4,5]. This requires oxidation of the methyl group by the Ten-eleven translocation (TET) family proteins and thymine-DNA glycosylase (TDG) [6]. Of note, TET proteins first hydroxylate 5-methylcytosine (5mC) to 5-hydroxymethylcytosine (5hmC) in mammals, which is a critical step in DNA demethylation. TET further oxidizes 5hmC to 5-formylcytosine (5fC) and 5-carboxylcytosine (5caC) [7,8], both of which can be excised by TDG, a component in the base excision repair (BER) machinery, eventually completing the cycle of DNA demethylation [9] (Figure 1).

In this review, we will focus on hypoxia-induced EMT and hypoxia-related epigenetic changes, particularly DNA demethylation mediated by TET proteins.

## 2. Hypoxia-Induced EMT

**Figure 2:**
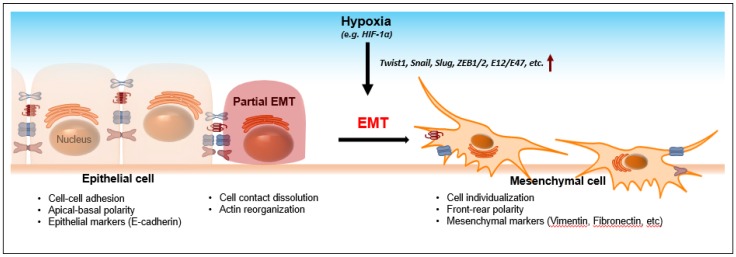
The epithelial-mesenchymal transition (EMT). The process of EMT requires loss of cell-cell contact, apical-basal polarity, and adhesion molecules (e.g., E-cadherin). Transformed epithelial cells then gain front-rear polarity and dissociate from the neighboring cells with increased expressions of mesenchymal markers. Thus, EMT increases cell motility which facilitates invasion and metastasis during cancer progression. Hypoxia is one of the driving forces that facilitate EMT. During hypoxia, many transcriptional regulators that govern the expressions of E-cadherin are activated by HIF-1α. As a result, downregulation of E-cadherin facilitates the development of mesenchymal phenotypes, leading to invasion and metastasis.

The epithelial-mesenchymal transition (EMT) is characterized by loss of cell-cell adhesion and apical-basal polarity. During EMT, epithelial cells lose E-cadherin expression and obtain mesenchymal markers, such as vimentin and fibronectin [10]. Subsequently, rearrangement of the cytoskeleton and cell-cell dissociation endows transited cells with a mesenchymal phenotype, such as increased cell protrusions and motility. EMT has been known to be an important process during embryonic development as well as tumor progression. For the latter, EMT promotes invasion, which is considered an initial and critical step for metastasis (Figure 2). E-cadherin loss, as a hallmark of EMT, is therefore a diagnostic biomarker in many cancers, including head and neck cancer [11], breast cancer [12], pancreatic cancer [13], *etc.*

Hypoxia upregulates the transcription factor, hypoxia-inducing factor (HIF-1α and HIF-2α), which, in turn, modulates a variety of target gene expressions in metabolism [14,15,16], tumor growth [17], EMT and metastasis [18,19], angiogenesis [20], and stemness [21,22]. In particular, hypoxia/HIF-induced EMT is a well-known phenomenon which has been implicated in numerous cancers [23]. Several EMT transcriptional regulators, including Twist1, Snail, Slug, ZEB1/2, and E12/E47, are activated either transcriptionally directly or indirectly through HIF-1α under hypoxia [19]. Subsequently, these EMT regulators bind to the promoters of EMT marker genes, such as E-cadherin, *etc.*, to facilitate EMT [24]. Aberrant expressions of HIF-1α, EMT inducers, and E-cadherin are correlated with one another and are associated with lymph node metastasis, an advanced TNM stage, and shorter patient survival [25,26]. Given the fact that hypoxia-mediated EMT is pivotal in cancer progression and in patients’ clinical outcomes, it would be paramount to explore the molecular mechanism that leads to tumor aggressiveness.

## 3. Epigenetic Alterations under Hypoxia

Hypoxia can induce epigenetic alterations at chromatin remodeling or DNA demethylation levels. Chen *et al.* have found that H3K9me2 is induced by histone methyltransferase G9a both at the global and gene-specific levels under hypoxia [27]. In support of this, using ChIP-chip and RNA expression profiling at a genome-wide scale, Xia *et al.* have defined HIF-1 chromatin binding targets, including jumonji-domain histone demethylases (JHDMs or KDMs), whose upregulation maintains histone methylation homeostasis, especially at the sites of H3 lysine 4, 9, and 36 [28]. Hypoxia regulates chromatin modifiers, such as histone lysine-specific demethylase 2B (JMJD2B or KDM4B), whose expressions correlate with the advance of colorectal cancers [29]. Upregulation of JMJD2B during hypoxia results in decreased H3K9me3 levels on the promoters of a subset of hypoxia-regulated target genes [29]. Its related demethylase, JMJD2C (*i.e.*, KDM4C), is found to be upregulated by HIF-1α and it can serve as a co-activator for HIF-1α which epigenetically modulates metabolic reprogramming and metastasis in breast cancer cells by decreasing H3K9me3 as well [30]. Other well-known hypoxia-induced epigenetic modifications involve histone deacetylases (HDACs). Wu *et al.* have found that HIF-1-activated HDAC3 downregulates H3K4Ac on the one hand and interacts with WDR5 and histone methyltransferase (HMT) complex to increase H3K4me2/me3 levels on the other [31]. This finding demonstrates that a crosstalk between coregulators (HDAC3 *vs.* HMT complex) under hypoxia can together modulate specific histone marks (*i.e.*, H3K4me2/me3) and hence mediates hypoxia-induced EMT [32]. For instance, HIF, p300, and HDAC4, HDAC5, or HDAC7 can form a multiprotein complex, thereby enhancing the expressions of HIF-regulated target genes [33,34]. Hypoxia also influences ATP-dependent chromatin remodeling complexes to restructure nucleosomes. The SWI/SNF chromatin remodeling complex can associate with HIF-1 and is required for HIF-1 transactivation [35]. Knockdown of SWI/SNF components reduces HIF-1α mRNA, indicating HIF-1 itself is a direct target of the SWI/SNF complex under hypoxia and there exists a positive feedback loop [35].

## 4. Detection of DNA Methylation Status under Hypoxia

Besides these histone changes, studies on epigenetic modification have been extended to DNA methylation/demethylation. Feinberg and Vogelstein first reported global DNA hypomethylation in the colorectal cell lines and patient specimens [36]. Over the past decades, numerous studies have been conducted to elucidate the modifications of DNA patterning at the genomic level. Methylome at single-base resolution has provided copious information on DNA composition in both coding and non-coding regions of the genome in embryonic stem cells, *etc.* [37]. To investigate global DNA methylation alteration, Shahrzad *et al.* have identified DNA hypomethylation during hypoxia by examining the amount of 5mC by HPLC in colorectal and melanoma cancers [38]. These epigenetic changes correlate with the severity of cancers not only in cell lines, but also in a xenograft model where an inverse relation is present between the magnitude of hypoxia and a reduction of 5mC [38]. Pal *et al.* have measured the DNA methylation status of short interspersed nuclear elements (SINEs), *i.e.*, *Alu*, and long interspersed nuclear elements (LINEs), as they are known to contribute to genome instability during hypoxia [39]. Using bisulfite sequencing to measure methylation and real-time PCR and inter-*Alu* PCR to quantify the transcripts of SINEs and LINEs, they have found that long-term hypoxic stress causes hypomethylation at these repetitive regions in glial tumor and osteosarcoma [39]. Recently, Liu *et al.* have found that hypoxia can induce global DNA demethylation by transcriptionally upregulating methionine adenosyltransferase II, alpha (MAT2A) in human hepatoma cells, maintaining the S-adenosylmethionine (SAM)/S-adenosylhomocysteine (SAH) ratio at a low level [40]. However, contrary to the observations in these aggressive tumors, the relation between hypoxia and DNA hypermethylation has been detected in normal tissues [41]. Prolonged ischemia causes cardiac fibrosis and the hypoxia-induced pro-fibrotic phenotype is associated with global DNA hypermethylation, and increased DNMT1 and DNMT3B expressions [41]. Similarly, in benign prostate PwR-1E epithelial cells, chronic hypoxia also increases DNA methylation and H3K9 acetylation [42]. Both discoveries use non-cancerous types of cells exposed to a long period of hypoxia, which may partly explain why the results are opposite to the findings reported in cancers. Also, different methods were utilized to obtain DNA composition. Where SINEs and 5mC were used to determine methylation in cancer cell lines, Watson *et al.* used flow cytometry to analyze average levels of DNA methylation per PwR-1E cell. It is worth mentioning that conventional bisulfite sequencing may misinterpret the cytosine information because C/5fC/5caC all react with sodium bisulfite and are deaminated to uracil (C/5fC) or 5caU (5caC), and are later sequenced as thymine (T), whereas 5mC and 5hmC are sequenced as C. Therefore, a more sensitive sequencing technique, such as methylase-assisted bisulfite sequencing (MAB-seq) as well as other base-resolution mapping methods, e.g., TET-associated bisulfite sequencing (TAB-seq), should be conducted to provide genome-wide quantitative information of cytosine states with single-based resolution [43]. Nonetheless, hypoxia-adapted cells require specific gene expressions and their upregulations are accompanied by the change of epigenetic profiling.

Epigenetic modification at a single gene can correlate with the advanced stage of tumors. In gastric cancers, the mRNA of a proto-oncogene, synuclein gamma (SNCG), is highly expressed due to its CpG demethylation whereas its expression is not detected in non-neoplastic gastric mucosal tissues [44]. Of the primary cancers tested, SNCG demethylation has a higher correlation with lymph node metastasis and advanced stage than those without lymph node involvement or in early stage, respectively [44]. Overexpression of HIF-1α is frequently identified partly by CpG demethylation at its own promoter which harbors a hypoxia response element, resulting in auto-transactivation and self-amplification in colon cancer [45]. In line with hypoxia-induced tumor malignancy, DNA hypermethylation at the promoter of PHD3 and VHL, two enzymes involved in destabilization of the HIF-1α protein, is observed in multiple myeloma and B-cell lymphoma [46]. Their downregulations may sustain HIF-1 protein stability and favor HIF-1 transactivation, thus promoting B-cell neoplasia. In addition, colorectal carcinoma (CRC) cells which were subjected to hypoxia and hypoglycemia had reduced DNMT1, DNMT3a, and DNMT3b mRNAs, resulting in a decrease in the 5mC level at the proximal promoter region of p16INK4a [47]. These lines of evidence support the notion that epigenetic modification, whether global or site-specific DNA methylation, participates in hypoxia-induced tumor progression by regulating gene expressions required for aggressive phenotypes.

## 5. TET-Mediated Demethylation in Cancer

**Figure 3:**
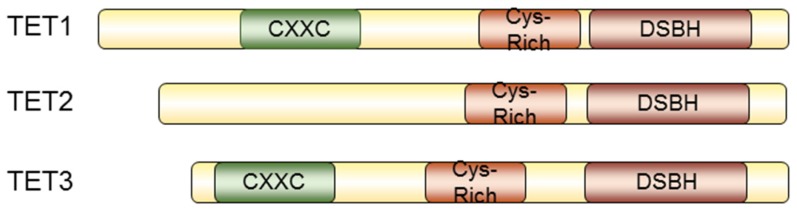
The TET protein family and their structures. Schematic structures of TET1, TET2, and TET3. TET proteins contain a Cys-rich region and a double-stranded helix (DSBH) at the C-terminus. TET proteins require cofactors, such as Fe(II) and 2OG, to hydroxylate substrates. Both TET1 and TET3 proteins contain a CXXC zinc-finger domain at the N-terminus, which can distinguish the methylation status of DNA.

TET proteins (TET1, TET2, and TET3) are mammalian homologs of the trypanosome proteins JBP1 and JBP2, and have other orthologs in metazoa, while homologous domains are found in fungi and algae as well [7]. As a 2OG-Fe(II) oxygenase, TET proteins contain a typical double-stranded helix (DSBH) fold at the C-terminus, with conserved residues for coordinating the cofactors Fe(II) and 2OG [7]. Vertebrate TET1 and TET3 also possess a CXXC-type zinc-binding domain, which is known to distinguish between methylated and unmethylated DNA [48] (Figure 3). It has been found that TET enzymes, as well as 5hmC, are highly expressed in various tissues, including primordial germ cells [49], Purkinje neurons [50], zygotes [51], and embryonic stem (ES) cells [52,53]. TET1 participates in ES maintenance and its expression correlates with the 5hmC levels [54,55,56,57,58]. TET1 binds to the transcription start sites (TSSs) of CpG-rich promoters and gene bodies of pluripotency factors in embryonic stem cells [54,55]. Intriguingly, Tet1 also binds to Polycomb-targeted developmental regulators and contributes to gene silencing [54,55]. Therefore, TET1 maintains pluripotency in embryonic stem cells by modulating DNA methylation on the one hand, and by transcriptionally repressing developmental regulators on the other. Recruitment of TET1 by NANOG is detected in mouse embryonic stem cells, whose presence increases 5hmC levels in a set of reprogramming target genes of NANOG to maintain pluripotency and lineage commitment [59]. TET1 also affects genomic imprinting in the paternal allele [9,60,61] and meiotic gene expressions in female germ cells [62,63]. Significance of TET3 is demonstrated by Gu *et al.* in their report where they have found a correlation between 5hmC levels and TET3 mRNA in the male pronucleus [64]. Knockout of TET3 abrogates paternal genome conversion of 5mC into 5hmC, abolishes demethylation of the paternal Oct4 and Nanog genes, and increases aberrant hydroxylation in the oocytes, therefore compromising embryonic development [64]. These data have established a correlation between TET proteins and embryonic development, where the level of 5hmC is a critical marker for cell fate determination.

**Figure 4:**
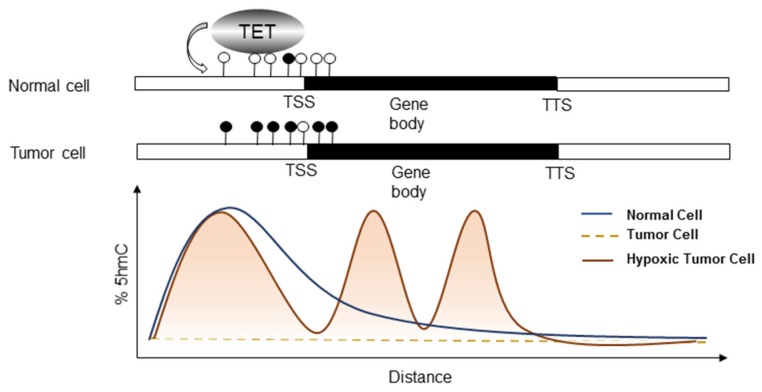
Methylation status and 5hmC levels in normal, tumor, and hypoxic tumor cells. In normal cells, particularly in ES and hematopoietic cells, TET proteins are highly abundant, usually in accordance with an enrichment in 5hmC and unmethylated DNA (white circle). However, during tumorigenesis, many of the CpG islands near or around the promoters of tumor suppressors are highly methylated (dark circle). Loss of 5hmC and TET proteins can be detected globally or site-specifically. However, when tumor cells undergo hypoxia, TET proteins can be induced by HIF-1α and elevated 5hmC composition can be detected at promoters, introns, exons, as well as 3′-UTR at a global scale (76), leading to gene expressions, e.g., those in the EMT and metabolic pathways. Hypoxic tumor cells are thus more malignant with enhanced migration/invasion, angiogenesis, and stemness, *etc.*, and resistant to anti-cancer drugs. TSS: transcriptional start site; TTS: transcriptional termination site.

Song *et al.* first used a chemical labeling technique to demonstrate the genome-wide distribution of 5hmC in human cell lines [65]. Later, loss of the TET activity as well as 5hmC contents is found to be associated with tumor development in certain solid tumors [66,67,68,69] (Figure 4). For example, colon cancer is characterized by loss of TET1. Expression of TET1 reduces cell proliferation by binding to the promoter of the DKK inhibitors of the WNT signaling pathway, keeping them hypomethylated and silenced [70]. Hsu *et al.* explored the function of TET1 in tumor invasion [71]. TET1 downregulation is found in prostate and breast cancer tissues, which facilitates tumor growth, cell invasion and metastasis. TET1 activates tissue inhibitors of metalloproteinase (TIMP) proteins 2 and 3 by inhibiting their DNA methylation. Low levels of TET1, TIMP2, and TIMP3 correlate with advanced stage in breast cancer patients [71]. Further investigation demonstrates that reduced TET proteins and TDG mRNAs are associated with poor prognosis in patients with early breast cancer [72].

Moran-Crusio *et al.* have delineated that conditional loss of TET2 in mice increases hematopoietic stem cell (HSC) self-renewal and myeloproliferation [73,74,75]. Studies using a large cohort of leukemia patients suggest frequent *TET2* mutations in myeloid malignancies and B- and T-cell lymphoma [73,74,76,77,78,79,80]. Recently, Wang *et al.* have found that Wilm’s tumor gene (WT1) is mutated in acute myeloid leukemia (AML) in a mutually exclusive manner with TET2 mutation [81]. TET2 binds and activates WT1 target genes by increasing the 5hmC levels at the promoter regions of these specific sites in normal HSC, and therefore TET2 inhibits leukemia proliferation in a WT1-dependent manner [81]. In addition to AML, the authors also pointed out that WT1 and TET2 genes are mutated in other types of tumors, including bladder, breast, kidney, liver, lung and uterine cancers [81]. It would be interesting to inquire whether TET2 suppresses tumor formation in these solid tumors in WT1-dependent or -independent pathways. However, Ko *et al.* have surprisingly found that low 5hmC is associated with hypomethylated CpG sites compared to the healthy controls in myeloid cancers with mutant TET2 [82], implying that TET2 might modulate DNA methylation indirectly via recruitment of other DNA methyltransferases. Experiments using co-immunoprecipitation of 5hmC-binding proteins should be tested to provide a concrete answer to this hypothesis. More recently, TET1 has been identified as a tumor suppressor in hematopoietic malignancy as well [83]. TET1-deficient tumors reveal mutations of non-Hodgkin B cell lymphoma (B-NHL), showing that TET1 is required for B cell lineage [83]. TET1 mutations in hematopoietic malignancy have a much lower frequency than TET2 mutations and their deletions cause different tumor types, suggesting that TET1 and TET2 are non-redundant and lineage-specific [83]. TET3 mutation has been reported in colon cancer, but its biological consequence in tumorigenesis has not been fully uncovered.

## 6. Bridging Hypoxia-Induced EMT to TET

**Figure 5:**
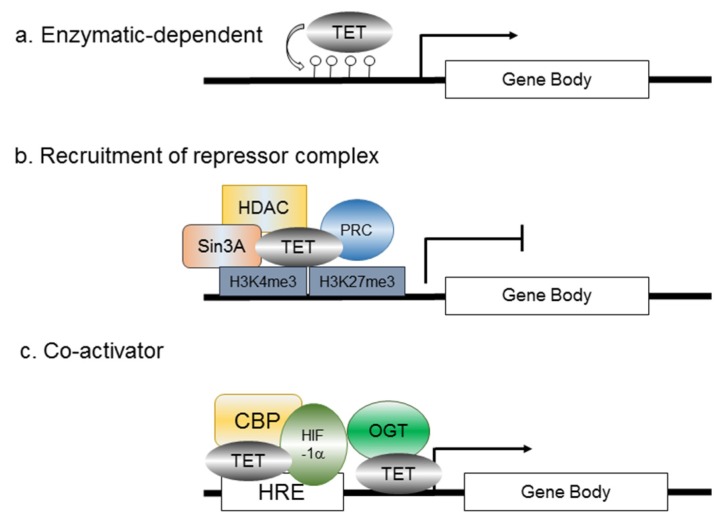
Mechanisms of TET-mediated transcription. (**a**) The canonical regulation of TET-mediated transcription relies on its dioxygenase activity, which turns 5mC into 5hmC; (**b**) A subset of TET proteins can recruit repressor complexes, such as PRC and Sin3A/HDAC, whereas a great proportion of TET proteins are found to bind to the CpG islands of TSS. As in the case of ES cells, TET enacts the dual functions by facilitating transcription of pluripotency factors via DNA demethylation and transcriptionally repressing developmental regulators; (**c**) TET proteins also act as a co-activator under hypoxia. TET proteins are associated with HIF-1α and CBP proteins at the HRE of a set of metabolic genes, e.g., *INSIG1*. TET can also interact with OGT at TSS. This leads to histone 2B Ser112 GlcNAc, and it further facilitates its monoubiquitination [91], presumably activating transcription. PRC: Polycomb repressive complex; HDAC: histone deacetylase; HRE: hypoxia-response element; OGT: O-GlcNAc transferase.

Interestingly, although TET activities and 5hmC loss are frequently observed in cell lines or tumor specimens, tumors exposed to hypoxia are associated with an upregulation of global 5hmC by TET induction [84]. The Godley group shows that hypoxia can induce TET1 expression, thereby enriching global 5hmC, and 5hmC is specifically gained at hypoxia-regulated genes in neuroblastoma [84] (Figure 5a). The versatile role of TET proteins in transcription regulation independent of their enzymatic activity has been reported in other studies. TET1 can bind to H3K4me3- and H3K27me3-enriched promoters and recruit the EZh2 Polycomb complex indirectly [54], or it can associate with the Sin3a histone deacetylase repressive complex [55] (Figure 5b). Moreover, proteomic studies have revealed binding between TET2, TET3 and the O-linked N-acetylglucosamine transferase (OGT) glycosyltransferase [85]. TET2/3 promotes GlcNAcylation and enhances H3K4me3 via SET1/COMPASS methyltransferase complex [86]. These findings may link metabolism to epigenetic modification, and to the transcriptional control of gene expression. In agreement with the above discoveries where TET mediates transcriptional regulation independent of the dioxygenase activity, Tsai *et al.* further explore the role of TET1 under hypoxia [87]. By RNA sequencing and 5hmC sequencing comparing TET1 knockdown cells under normoxia with those under hypoxia, one of the regulators in the cholesterol metabolic process, insulin induced gene 1 (INSIG1), was identified. Among other affected genes are mostly members in metabolic pathways, including sterol and farnesyl diphosphate metabolism, suggesting a tight connection between epigenetic modification and metabolic processing. Knockdown of TET1 or INSIG1 mitigates the expression of a set of hypoxia-induced genes involved in glucose metabolism and EMT. Interestingly, TET1 not only increases 5hmC peaks in the INSIG1 promoter, but also serves as a transcription co-activator independent of its enzymatic activity to modulate the transactivation activity of HIF-1α [87]. TET1 may form a complex with HIF-1α/CBP, or with OGT, to facilitate hypoxia-mediated gene expressions [87] (Figure 5c). This line of evidence first connects hypoxia-regulated TET1/5hmC to metabolism and EMT. TET2 also recruits Hdac2 to repress IL-6 gene expression independent of its DNA demethylating activity [88]. In line with this investigation, Wu *et al.* have also demonstrated that in breast cancer, hypoxia/HIF causes genome-wide changes in DNA demethylation by upregulating TET1 and TET3, which, in turn, mediate the activation of TNFα-p38-MAPK [89]. This pathway is essential for breast tumor initiating cell (BTIC) capabilities as well as EMT promotion in breast cancer, suggesting a linkage between hypoxia-induced EMT and TET proteins. Other example shows that TET1 knockdown suppresses their proliferation and blocks cell-cycle progression at the G1 phase in human 786-O renal cells [90]. Further investigation is required to determine the impact of hypoxia-regulated TET1 and its possible role in EMT/metabolism in cancer.

Undoubtedly, TET proteins shall participate in hypoxia-mediated hypomethylation and lead to enhanced malignancy. Cancer-associated genome-wide maps of methylation must be systemically established to evaluate the correlation between TET proteins and cancer progression.

## 7. Hypoxia-Activated Prodrugs (HAPs) as a Potential Therapeutic Approach

TET2 mutation or inactivation suggest that perturbation of DNA methylation may cause hematopoietic malignancy. Targeting TET proteins as a therapeutic method is thus less favorable due to their inactivation status in most cancers. Imbalance in DNA methylation, *i.e.*, hypermethylation of tumor suppressor genes in cancers, can be corrected by hypomethylating agents, such as 5-azacitidine and decitabine [92,93], and their applicability has already been tested in clinical studies. However, these drugs are non-specific and their long-term effect is still unknown [94]. Although TET1 is upregulated by hypoxia, its clinical implication has not been reported yet. On the contrary, hypoxia itself is a common and persistent feature within solid tumors, either chronically or acutely, and it serves as a negative prognostic factor in numerous clinical studies [95]. Its prevalence impedes treatment failure due to inaccessibility by most anti-cancer agents to these chronic hypoxic regions [96]. Moreover, hypoxic cells tend to be more quiescent in the cell cycle, reducing the efficacy of most anti-cancer drugs, which target proliferating cells [97]. They are less sensitive to apoptosis and can upregulate drug resistance proteins as well [98]. In radiation-resistant cancer cells, lowered oxygen levels reduce the formation of peroxide radicals, undermining the effectiveness of radiation [99]. Numerous attempts have been made to tackle hypoxic tumor cells with respect to therapeutic agent development. Among them, hypoxia-activated prodrugs (HAPs) are rising candidates in cancer therapy. Mechanistically, HAPs remain reduced and active drugs can be produced by further reduction in hypoxic cells whereas sufficient oxygen in normal cells oxidizes the chemicals back to the prodrug state. Five chemical moieties, *i.e.*, nitro groups, quinones, aromatic and aliphatic N-oxides, and transition metals, can be designed to be chemically reduced under hypoxia [100]. These backbones give diverse sensitivity to oxygen concentrations and those activated under extremely hypoxic conditions have been designed to possess greater local diffusion ability to neighboring cells, causing the bystander effect and killing cells at higher oxygen concentration [101]. A detailed review on these HAPs can be found in [101]. Given the fact that TET proteins are upregulated under hypoxia, HAPs may possibly alter aberrant DNA demethylation, relieving pro-tumorigenic effects induced by hypoxia, including metabolic adaptation, immune suppression, and promotion of EMT.

One should notice, however, that enzymes are required to catalyze one-electron transfer to prodrugs. Therefore, profiling and identification of these enzymes in individual tumors are of top priority if HAPs are to be used [101]. Moreover, sometimes there is poor correlation between different hypoxia markers in preclinical and clinical cases. Selecting suitable patients for a specific HAP requires adequate diagnostic tools.

## 8. Perspectives

DNA methylation is a critical modulator in gene silencing and its presence propagates hereditary messages during development. This epigenetic mark is reversible and can be actively processed by a class of dioxygenase, TET family proteins. TET proteins generate 5hmC, a hallmark of DNA demethylation. Aberrant epigenetic modification occurs in cancer cells. Loss or repression of TET protein functions and 5hmC can be found in leukemia and certain solid tumors. Recent studies have identified TET proteins as the mediators that modulate hypoxia-induced 5hmC contents. Global demethylation facilitates expressions of pro-tumorigenic genes, especially those in the metabolic pathways, e.g., INSIG1, which, in turn, enhances hypoxia-induced EMT. Hypoxia poses an impediment against most anti-cancer drugs for several reasons (see Section 7). HAPs can specifically target hypoxic cells yet diffuse to the neighboring cells to eradicate cancer cells. Preclinical as well as clinical trials of HAPs have been conducted in combination with other chemotherapy. Hopefully, these therapeutic strategies may eliminate tumor cells and serve as a standard treatment in clinical care options.
